# Facile Electrochemical Synthesis of Bifunctional Needle-like Co-P Nanoarray for Efficient Overall Water Splitting

**DOI:** 10.3390/molecules28166101

**Published:** 2023-08-17

**Authors:** Xiong He, Jiayang Cai, Jie Zhou, Qiyi Chen, Qijun Zhong, Jinghua Liu, Zijun Sun, Dezhi Qu, Yudong Li

**Affiliations:** 1 Liuzhou Key Laboratory of New Energy Vehicle Power Lithium Battery, School of Electronic Engineering, Guangxi Engineering Research Center for Characteristic Metallic Powder Materials, Guangxi University of Science and Technology, Liuzhou 545000, China; hexiong@gxust.edu.cn (X.H.); 17586600924@163.com (J.Z.); 15296323062@163.com (Q.C.); zqj17878906682@163.com (Q.Z.); sunzijun@gxust.edu.cn (Z.S.); 2 Guangxi Key Laboratory of Green Processing of Sugar Resources, College of Biological and Chemical Engineering, Guangxi University of Science and Technology, Liuzhou 545006, China; cjy193677464@163.com (J.C.); qudezhi199166@gxust.edu.cn (D.Q.); 3 Key Laboratory of Bio-Based Material Science & Technology, Northeast Forestry University, Harbin 150090, China

**Keywords:** electrodeposition, overall water splitting, cobalt phosphide, needle-like structure

## Abstract

The development of low-cost and high-performance bifunctional electrocatalysts for overall water splitting is still challenging. Herein, we employed a facile electrodeposition method to prepare bifunctional cobalt phosphide for overall water splitting. The needle-like cobalt phosphide (Co-P-1) nanoarray is uniformly distributed on nickel foam. Co-P-1 exhibits excellent electrocatalytic activity for hydrogen evolution reaction (HER, 85 mV at 10 mA/cm^2^, 60 mV/dec) and oxygen evolution reaction (OER, 294 mV at 50 mA/cm^2^, 60 mV/dec). The cell-voltage of 1.60 V is found to achieve the current density of 10 mA/cm^2^ for overall water splitting in the two-electrode system, comparable to that of previously reported Pt/C/NF||RuO_2_/NF. The excellent electrocatalytic performance can be attributed to the needle-like structure with more active sites, accelerated charge transfer and evolved bubbles’ release. This work can provide new approach to the development of a bifunctional electrocatalyst for overall water splitting.

## 1. Introduction

With the excessive consumption of fossil energy and the ensuing environmental issues, the development of clean energy is urgently demanded [1,2]. Hydrogen with zero carbon emissions, high calorific value, and easy storage and transportation has attracted much attention [3]. Electrocatalytic water splitting is considered as one of the most green and facile approaches for hydrogen production [4]. Electrocatalytic water splitting consists of a hydrogen evolution reaction (HER) and oxygen evolution reaction (OER) with the theoretical overpotential of 1.23 V. However, the sluggish kinetics of OER and HER restrict hydrogen production efficiency of water splitting. The high overpotential and energy barrier make it necessary to explore efficient electrocatalysts for water splitting. Even though the noble-metal-based catalysts possess excellent catalytic performance for OER and HER, the scarcity and cost limit their large-scale industrial application [5,6,7]. Meanwhile, bifunctional catalysts for both OER and HER are beneficial for diminishing the complexity of catalytic setup, simplifying the design and fabrication of the whole system, and reducing cost [8,9]. But, most electrocatalysts present only a single catalytic activity for OER and HER, resulting in the increasing complexity of overall water splitting technology [10]. Thus, the exploration and development of efficient and low-cost bifunctional electrocatalysts are meaningful and challenging.

Recently, transition metal phosphides, especially for cobalt phosphides, are considered to be promising bifunctional electrocatalysts for efficient water splitting owing to their high electron conductivity and thermal stability resulting from their orthogonal structure [11]. Cobalt phosphides were reported to be highly active for HER in acidic, alkaline and neutral media [12,13]. The phosphorous sites are considered to be significant for HER because the electronegative P can attract positively charged protons during the HER process [14]. In addition, the metal-rich phosphides can exhibit the metallic character with an appreciable difference in electronegativity [13]. The regulated electronic structure and the decreased hydrogen adsorption energy barrier with P site could accelerate HER performance [15]. Kim et al. showed that electrodeposited Co-P possesses excellent HER performance due to its superior electrochemical surface area (ECSA) and charge transfer characteristics [16]. Meanwhile, transition metal phosphides are regarded as pre-catalysts with the phase transformation from transition metal phosphides to active transition metal oxyhydroxides during the OER process [17]. The OER active sites which are believed to be high-valence metal sites are exposed during the in situ electrochemical transformation under applied anodic potential [18]. Sheng et al. reported that highly active NiCoP is reconstructed into Ni(Co)OOH as real active sites with P element loss during OER [19]. Additionally, the one-dimensional catalysts favor the acceleration of charge transfer and expose more active sites for enhancing OER and HER performance [20]. And the integrated nanoarray catalysts possess the advantages of structural stability, high surface area with more active sites, and facilitated electron transfer [21]. For example, a phosphorus-doped Co_3_O_4_ nanowire array has an efficient OER and HER performance (260 mV at 20 mA/cm^2^ with 60 mV/dec for OER, and 97 mV at 10 mA/cm^2^ with 86 mV/dec for HER), and exhibits a potential of 1.63 V to achieve an overall water splitting current of 10 mA/cm^2^ in a two-electrode system [22]. A CoP nanoarray grown on titanium mesh displays bifunctional OER and HER activity, and a cell voltage of 1.70 V is required to obtain 15 mA/cm^2^ [23]. There are various preparation methods of cobalt phosphides, such as hydrothermal [24], thermal decomposition [25], chemical vapor deposition (CVD) [26], and temperature-programmed reduction (TPR) [27]. Compared with hydrothermal, CVD, TPR, and thermal decomposition methods, the electrodeposition of cobalt phosphides is direct and fast, which is more suitable for large-scale industrial application. However, to our knowledge, the direct preparation of a needle-like Co-P nanoarray with the electrodeposition method has not been previously reported.

Herein, we report a facile electrodeposition of a needle-like Co-P (Co-P-1) nanoarray on nickel foam as a bifunctional electrocatalyst for OER and HER. The needle-like structure could provide more active sites, accelerate charge transfer and promote evolved bubbles’ release. Hence, the needle-like Co-P-1 nanoarray exhibits excellent OER and HER electrocatalytic activity and stability. For HER, Co-P-1 expresses a low overpotential of 85 mV and Tafel slope of 60 mV/dec to deliver 10 mA/cm^2^ without obvious surface structure change. At the same time, Co-P-1 demonstrates good OER activity with an overpotential of 294 mV to deliver 50 mA/cm^2^, along with the transformation into CoOOH. Co-P-1 also displays good long-term stability of 24 h for both OER and HER. The cell voltage of 1.60 V is found to achieve the current density of 10 mA/cm^2^ for overall water splitting in the two-electrode system, comparable to the reported Pt/C/NF||RuO_2_/NF. This work supplies a novel approach for efficient bifunctional electrocatalyzation for overall water splitting.

## 2. Results and Discussion

### 2.1. Characterization of Prepared Catalysts

**Figure 1:**
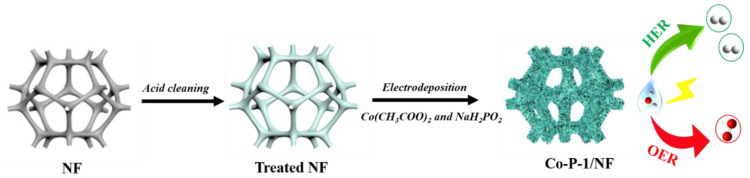
Schematic preparation diagram of Co-P-1/NF.

A needle-like Co-P-1 nanoarray was prepared via a facile electrodeposition method, as schematically presented in Figure 1. The pre-treated NF is applied as substrate, and a mixed aqueous solution containing 0.1 M cobaltous acetate and 0.5 M sodium hypophosphite is used as electrolyte. The Co^2+^, H_2_PO_2_^−^ and CH_3_COO^−^ are accumulated on the NF surface under the applied potential. Therefore, Co-P-1 can be formed with metal and phosphorus deposition based on the reduction of Co^2+^ and H_2_PO_2_^−^ [28]. For comparison, Co(OH)_2_ is electrodeposited on the NF surface without sodium hypophosphite addition. And cobalt phosphide (marked as Co-P-2) is further prepared with Co(OH)_2_ as precursor by thermal decomposition of sodium hypophosphite. The corresponding synthesis details can be found in the Materials and Methods section.

**Figure 2:**
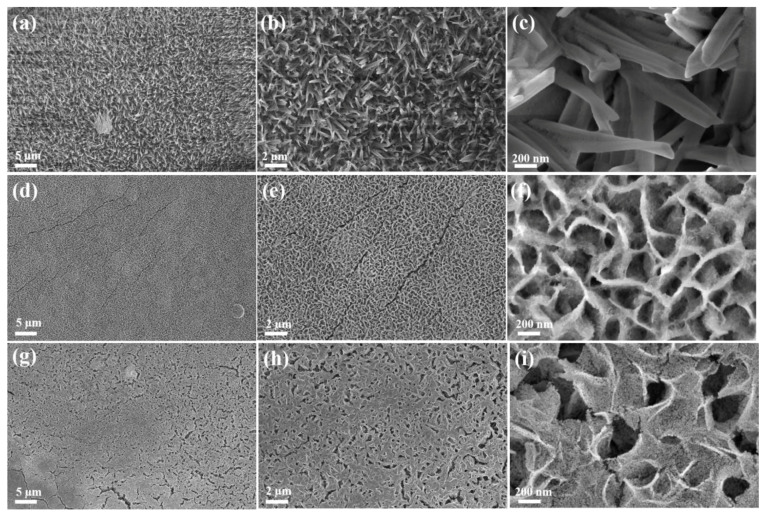
SEM images of (**a**–**c**) Co-P-1, (**d**–**f**) Co(OH)_2_, and (**g**–**i**) Co-P-2.

The morphologies of as-prepared Co-P-1, Co(OH)_2_, and Co-P-2 were investigated via scanning electron microscopy (SEM). As shown in Figure 2a–c, it can be observed that the needle-like Co-P-1 nanoarray is uniformly distributed on NF substrate. The length of Co-P-1 nanoneedles ranges from 1 to 2 µm. This needle-like structure of Co-P-1 may facilitate bubble release and electron transfer [29]. Meanwhile, the Co(OH)_2_ nanosheet array is uniformly distributed on the NF surface, as seen in Figure 2d–f. Co(OH)_2_ demonstrates a nanosheet thickness at 10–20 nm and roughened surface. After further thermal decomposition of sodium hypophosphite, Co(OH)_2_ is transformed into Co-P-2. As shown in Figure 2g–i, Co-P-2 still maintains a nanosheet structure. In addition, plentiful nanoparticles appear on the surface of nanosheets, which may be ascribed to the aggregation during thermal decomposition. During thermal decomposition, the generated PH_3_ can etch transition metal hydroxides and convert to phosphides, leading to the decreasing nanosheet thickness [30]. This phenomenon can be observed in comparison with Figure 2f,i.

**Figure 3:**
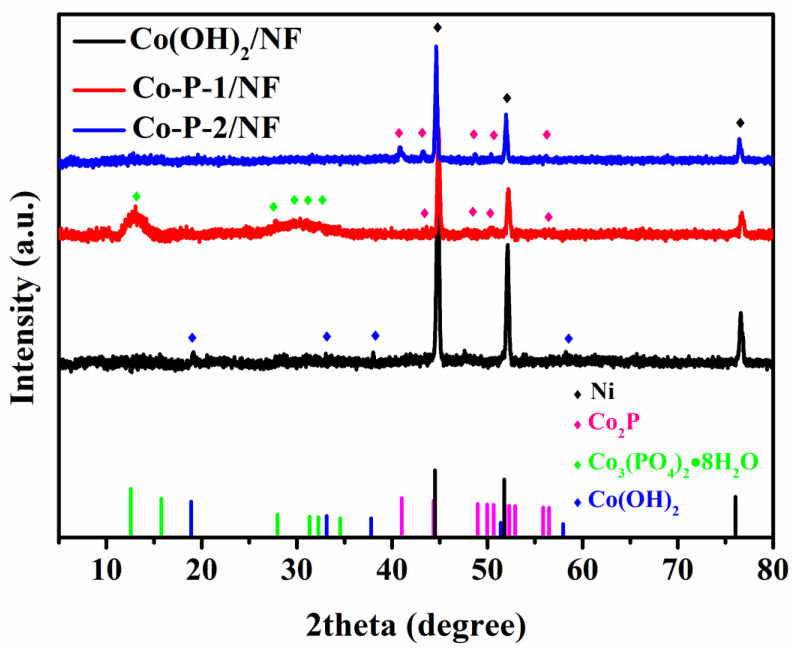
XRD patterns of Co(OH)_2_/NF, Co-P-1/NF, and Co-P-2/NF.

Figure 3 demonstrate the XRD patterns of Co(OH)_2_, Co-P-1, and Co-P-2. From these XRD patterns, three sharp Bragg peaks (2θ at 44.8, 52.1, and 76.6°) fitted with nickel (JCPDS No 04-0850, marked by black diamond) can be detected, which is originated from NF substrate. An amorphous structure can be obviously observed for these three samples. Amorphous structure is suggested to effectively enhance kinetics for HER/OER owing to the optimized adsorption energy of reaction intermediates and regulated electronic structure [10]. The crystalline–amorphous interface is beneficial for electrocatalytic activity [31]. For Co(OH)_2_, it displays a typical Co(OH)_2_ phase (JCPDS No 51-1731, marked by a blue diamond), suggesting a nanosheet-like Co(OH)_2_ formation on NF. After the thermal decomposition of sodium hypophosphite, Co-P-2 exhibits peaks of a Co_2_P (JCPDS No 32-0306, marked by a red diamond) crystal structure. It expresses metal hydroxides that are converted into metal phosphide during thermal decomposition. At the same time, Co-P-1 demonstrates peaks of Co_3_(PO_4_)_2_·8H_2_O (JCPDS No 35-0109, marked by a green diamond) and Co_2_P (JCPDS No 32-0306), declaring the complex composition of Co-P-1 (as shown in Figure S1).

**Figure 4:**
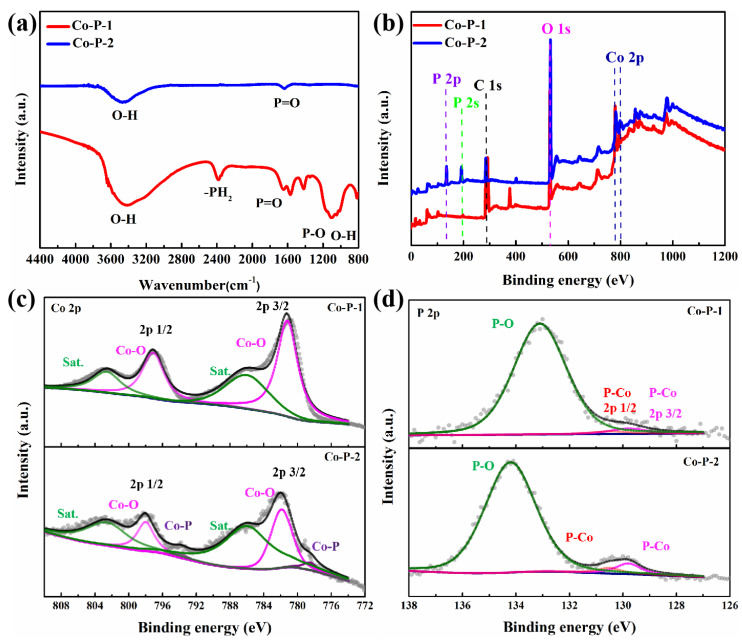
(**a**) FTIR spectra, (**b**) XPS survey, (**c**) Co 2p and (**d**) P 2p HR-XPS spectra of Co-P-1 and Co-P-2.

Fourier transform infrared spectroscopy (FTIR) spectra of Co-P-1 and Co-P-2 are displayed in Figure 4a. For Co-P-2, peaks at 3443 and 1641 cm^−1^ correspond to O-H stretching vibration and P=O stretching vibration, which may be ascribed to surface water adsorption and air oxidation [32]. For Co-P-1, the newly appeared peak at 2383 cm^−1^ corresponding to -PH_2_ stretching vibration can be observed. A wide peak at 1090 cm^−1^ is regarded as a combination peak of the P-O group and O-H group. Meanwhile, the coexistence of P-O, O-H, and -PH_2_ bonds express the presence of H_2_PO_2_^−^, which may have originated from the H_2_PO_2_^−^ intercalation [33]. And the existence of cobalt phosphate is confirmed from P=O and P-O peaks, in agreement with the XRD result. X-ray photoelectron spectroscopy (XPS) measurement was conducted to recognize the surficial chemical composition and electronic structure of electrocatalysts. The XPS survey spectra of Co-P-1 and Co-P-2 (Figure 4b) suggest the existence of Co, O, and P elements. The high-resolution (HR) Co 2p XPS spectra for Co-P-1 and Co-P-2 are displayed in Figure 4c. For Co-P-1, the fitted peaks at 781.2 and 797.1 eV are attributed to Co 2p 3/2 and Co 2p 1/2 for the Co-O bond with two satellite peaks at 786.0 and 802.6 eV. For Co-P-2, two extra peaks at 778.5 and 793.8 eV can be observed, corresponding to the Co 2p 3/2 and Co 2p 1/2 for Co-P bond. This result demonstrates successful phosphating with thermal decomposition. The P 2p HR-XPS spectra are exhibited in Figure 4d. For both Co-P-1 and Co-P-2, two peaks at 129.8 and 130.5 eV are originating from P 2p 1/2 and 2p 3/2 of P-Co bonds, suggesting the existence of cobalt phosphide. For Co-P-2, a wide peak at 134.2 eV can be obtained, which may be ascribed to the superficial oxidation of Co-P [34]. A main peak at 133.1 eV can be observed for Co-P-1, which may be ascribed to the P-O bond from H_2_PO_2_^−^, which is consistent with the FTIR result. These results illustrate the existence of H_2_PO_2_^−^, cobalt phosphate and cobalt phosphide on the surface of Co-P-1, which is active for HER/OER performance.

### 2.2. HER Performance

**Figure 5:**
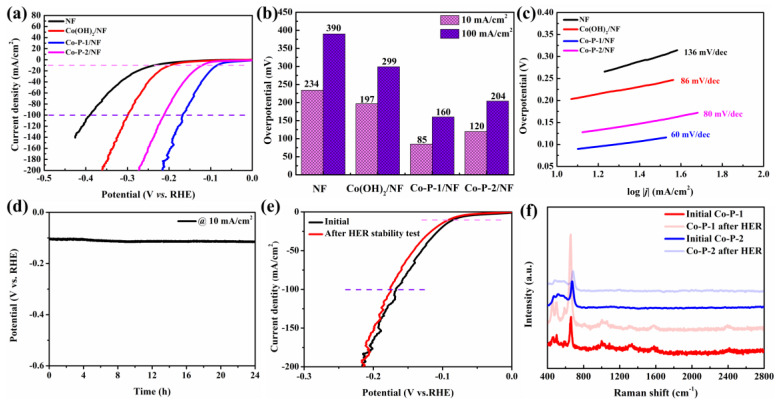
(**a**) The 80% *iR*-corrected HER polarization curves, (**b**) corresponding overpotentials at 10 and 100 mA/cm^2^, (**c**) corresponding Tafel plots, (**d**) CP curve of Co-P-1, (**e**) HER polarization curves of Co-P-1 before and after 24 h HER stability test, and (**f**) Raman of Co-P-1 and Co-P-2 before and after HER stability test.

To shed the ohmic resistance effect on andic current, the HER activities of these samples were explored by an 80% *iR*-corrected polarization test in 1 M KOH at a scan rate of 5 mV/s. The Co-P-1/NF exhibits excellent HER activity with lower overpotential (*η*) and higher current density than that of NF, Co(OH)_2_/NF, and Co-P-2/NF (Figure 5a). A similar result can be observed from polarization curves without *iR* correction (Figure S2a). In this context, the optimized Co-P-1 presents a small *η* of 85 mV to achieve a current density of 10 mA/cm^2^, which is superior to that of NF (234 mV), Co(OH)_2_/NF (197 mV), and Co-P-2/NF (120 mV) (Figure 5b). The value of *η* at 100 mA/cm^2^ demonstrates a similar trend. From Table S1, it can be observed that the Co-P-1 nanoneedle array exhibits superior HER performance to previous reports [16,19]. To gain insight into the catalytic HER performance in terms of the electron/mass transport and reaction kinetics, the Tafel slope was calculated from HER polarization curves. As exhibited in Figure 5c, the Tafel slope of Co-P-1/NF is found to be 60 mV/dec, which is lower than that of NF (136 mV/dec), Co(OH)_2_/NF (86 mV/dec), and Co-P-2/NF (80 mV/dec), indicating faster HER kinetics and better charge transfer ability. It may be ascribed to the fast charge transfer path provided by the unique needle-like structure. In addition, the Tafel slope can indicate the reaction pathway and rate-determination step. The Volmer step is the proton adsorption process, Tafel step is the physical desorption process, and Heyrovsky step is the chemical desorption process. The Tafel slopes of Volmer, Heyrovsky and Tafel reactions are calculated as 118.2, 39.4, and 29.6 mV/dec, respectively [35]. The Tafel slope of 60 mV/dec within the range from 39.4 to 118.2 mV/dec manifests that Co-P-1 follows the Volmer–Heyrovsky pathway and the rate-determination step is the Heyrovsky step [35,36]. The long-term stability of Co-P-1 for HER was measured by a chronopotentiometry (CP) test at 10 mA/cm^2^. In this regard, a small decay of 13% can be observed after a long-term working time of 24 h (Figure 5d). In addition, the LSV curves before and after the 24 h CP test were employed to evaluate the stability of Co-P-1. As shown in Figure 5e, no obvious change occurs in the comparison of LSV curves before and after the 24 h CP test, indicating good stability of the needle-like Co-P-1 nanoarray for HER. The Raman spectrum was conducted to investigate the composition change before and after HER test. For Co-P-2, Raman peaks at 479, 518, and 676 cm^−1^ correspond to the E_g_ and F_2g_ of the Co-O bond, and A_1g_ of Co^3+^, which are originated from air oxidation [37]. For Co-P-1, Raman peaks at 450, 496, and 584 cm^−1^ related to the Co-O bond of Co(OH)_2_ and cobalt phosphate can be obtained [38]. And, the extra Raman peaks at 1004 cm^−1^ originating from the A_1g_+E_g_ vibration of PO_4_^3−^ can be captured [37]. This indicates that cobalt phosphate is dominant in Co-P-1, in accordance with previous analysis. In comparison with Raman spectra of Co-P-1 and Co-P-2 before and after the HER test, the Raman peaks are nearly unchanged, suggesting that Co-P-1 and Co-P-2 keep their structure during the HER process.

### 2.3. OER Performance

**Figure 6:**
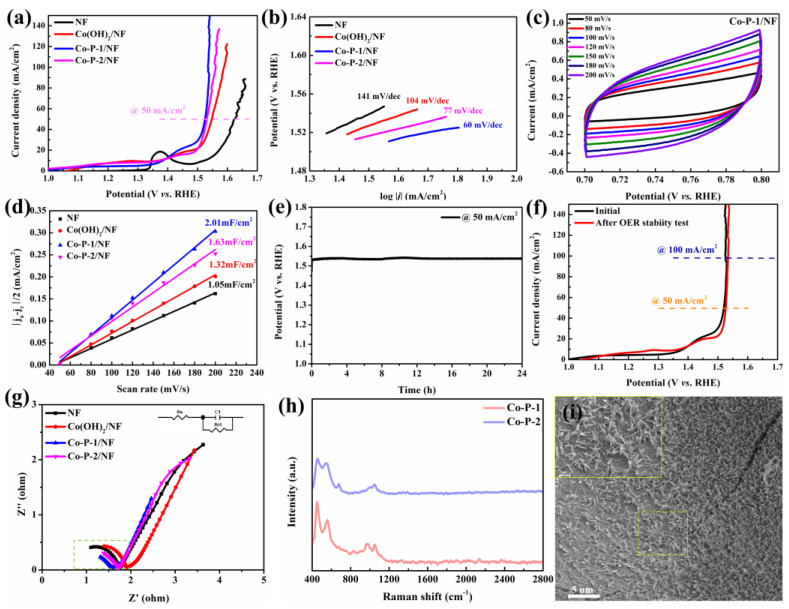
(**a**) The 100% *iR*-corrected OER polarization curves of these samples, (**b**) Tafel plots of these samples, (**c**) CV curves of Co-P-1/NF at various scan rates, (**d**) linear fitting of capacitive currents of these sample, (**e**) CP stability curve of Co-P-1 at 50 mA/cm^2^ for 24 h, (**f**) LSV curves before and after 24 h OER stability test, (**g**) Nyquist plots at 1.50 V, (**h**) Raman after OER test, and (**i**) SEM image of Co-P-1 after 24 h OER stability test.

The OER performance of electrocatalysts was evaluated with a three-electrode system in 1 M KOH. To fairly compare OER activity, 100% *iR*-corrected OER polarization curves of as-prepared catalysts are displayed in Figure 6a. It can be concluded that Co-P-1/NF requires a lower overpotential (294 mV) to reach 50 mA/cm^2^ than that of NF (394 mV), Co(OH)_2_/NF (317 mV), and Co-P-2/NF (301 mV). The polarization curves without *iR* correction exhibit a similar OER performance trend, as shown in Figure S2b. In comparison with previously reported electrocatalysts, the optimized Co-P-1/NF demonstrates comparable OER performance (see Table S2). The Tafel slope of Co-P-1 is calculated to be 60 mV/dec, which is smaller than those of NF (141 mV/dec), Co(OH)_2_/NF (104 mV/dec), and Co-P-2/NF (77 mV/dec), illustrating accelerated OER kinetics and more rapid charge transfer [39]. To evaluate ECSA, electrochemical double-layer capacitance (*C_dl_*) is measured with CV with various scan rates. The non-faradaic region is found from the CV curves of NF, Co(OH)_2_/NF, Co-P-1/NF, and Co-P-2/NF at a scan rate of 50 mV/s (Figure S3a). In this work, the potential region of 0.70–0.80 V vs. RHE was selected as the non-faradaic region. Figure 6c displays the CV curves of Co-P-1 in the potential region of 0.70–0.80 V vs. RHE at 50, 80, 100, 120, 150, 180, and 200 mV/s. The corresponding CV curves of NF, Co(OH)_2_/NF, and Co-P-2/NF are presented in Figure S3b–d. The quasi-rectangle forms also suggest the non-faradaic region for these electrocatalysts. The capacitive current increases with the scan rate. The linear fitting of capacitive currents of these samples are exhibited in Figure 6d. The *C_dl_* values for NF, Co(OH)_2_/NF, Co-P-1/NF, and Co-P-2/NF are calculated to be 1.05, 1.32, 2.01, and 1.63 mF/cm^2^, respectively. The result suggests a higher ECSA and more active sites for Co-P-1/NF, which may be related to the needle-like structure [40]. The long-term stability of Co-P-1/NF was measured with CP, as shown in Figure 6e. Co-P-1/NF shows good stability with potential increasing only 7 mV after 24 h at 50 mA/cm^2^. The OER polarization curves of Co-P-1/NF before and after the 24 h stability test are exhibited in Figure 6f. No obvious change occurs after the stability test, confirming the good stability of Co-P-1. The electrical properties of these catalysts were further investigated by EIS measurement. The Nyquist plots measured at 1.50 V vs. RHE are shown in Figure 6g. The semi-circles in Nyquist plots are fitted with the inserted equivalent circuit as presented in Figure S4. The fitted parameters were summarized in Table S3. The charge resistance of Co-P-1 was found to be 0.41 Ω, smaller than other catalysts (0.85 Ω for NF, 0.83 Ω for Co(OH)_2_/NF, and 0.54 Ω for Co-P-2/NF), suggesting faster charge transfer [18]. This result is associated with needle-like structure for more active sites, fast charge transfer and accelerated bubble release. Furthermore, the morphology and structure of Co-P-1 after the long-term OER test has been investigated. The Raman spectra of Co-P-1 and Co-P-2 after OER are provided in Figure 6h. The Raman peaks at 454 and 554 cm^−1^ indexed to CoOOH can be observed, indicating the transformation into CoOOH during the OER process. Meanwhile, Raman peaks at 973 and 1050 cm^−1^ can be obtained, which may come from the phosphate ligands [37]. These results manifest the phase transform to CoOOH with PO_4_^3-^ generation during the OER process. In addition, the morphology of Co-P-1 after the OER test is explored with SEM imaging. As shown in Figure 6i, the needle nanoarray is maintained after OER. From Figure S5, the needle structure is almost unchanged, expressing excellent stability of Co-P-1 during the OER process.

### 2.4. Overall Water Splitting

**Figure 7:**
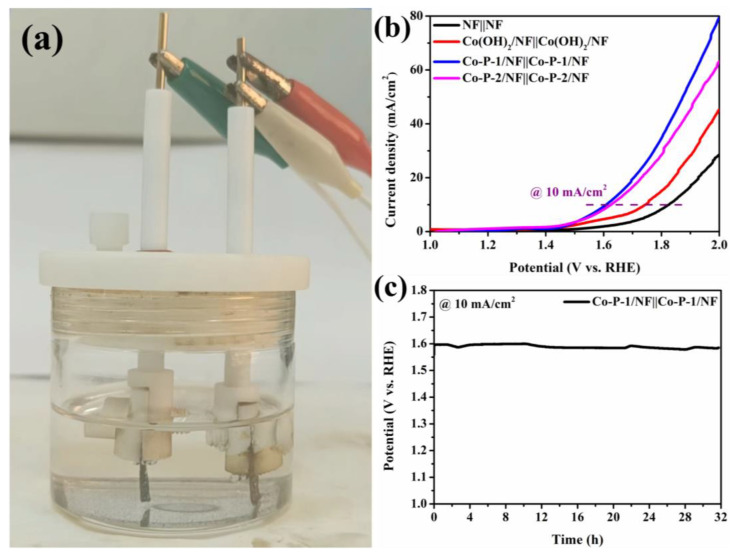
(**a**) Photograph of water-splitting device, (**b**) polarization curves of overall water splitting, and (**c**) long-term stability of Co-P-1||Co-P-1.

The good catalytic activities of Co-P-1 for both OER and HER indicate that it can be an efficient bifunctional electrocatalyst for overall water splitting. Figure 7a displays a photograph of the two-electrode device for overall water splitting. The corresponding polarization curves for overall water splitting are exhibited in Figure 7b. Co-P-1/NF||Co-P-1/NF exhibits optimum electrocatalytic activity for overall water splitting compared with NF||NF, Co(OH)_2_/NF||Co(OH)_2_/NF, and Co-P-2/NF||Co-P-2/NF. It can be found that the potential of 1.60 V to receive 10 mA/cm^2^ is required for Co-P-1/NF||Co-P-1/NF, which is comparable to that of the reported Pt/C/NF||RuO_2_/NF [10]. As shown in Figure 7c, the long-term stability of Co-P-1/NF||Co-P-1/NF is tested with CP measurement. Almost no decay is recognized after the 32 h test, confirming its superior stability. Therefore, the bifunctional needle-like Co-P-1 is believed to be a promising electrocatalyst for overall water splitting.

## 3. Materials and Methods

### 3.1. Reagents

Nickel foam (NF, 250 mm × 1000 mm × 1 mm, 95% porosity) was purchased from Taiyuan Lizhiyuan Technology Co., LTD (Taiyuan, China). Cobalt acetate (Co(CH_3_COO)_2_, 98%), ethanol (CH_3_CH_2_OH, 99.5%) and sodium hypophosphite (NaH_2_PO_2_, 99%) were purchased from Macklin. Pure water was home-made in the laboratory. All reagents were directly used without further purification.

### 3.2. Preparation of Co-P-1

Before electrodeposition, NF was cut into pieces of 1.5 × 0.5 cm^2^, and then washed with dilute hydrochloric acid, ethanol, and pure water two times. The electrodeposition process was conducted via a three-electrode system with a Ag/AgCl electrode as a reference electrode and Pt plate as the counter electrode at 25 °C. Due to the near-neutral electrolyte, the Ag/AgCl electrode was selected as the reference electrode. The aqueous electrolyte consisted of 0.1 M cobaltous acetate, and 0.5 M sodium hypophosphite. Electrodeposition was employed in a glass cell containing 30 mL electrolyte at a constant potential of −1.2 V vs. Ag/AgCl for 900 s. After washing with pure water, the needle-like Co-P-1 nanoarray grown on NF was prepared, which is donated as Co-P-1/NF.

### 3.3. Preparation of Co(OH)_2_ and Co-P-2

For comparison, cobalt hydroxide was electrodeposited on NF from an electrolyte without NaH_2_PO_2_ addition. Cobalt hydroxide was further phosphate with thermal decomposition of sodium hypophosphite as a phosphorous source. Some 0.5 g sodium hypophosphite and Co(OH)_2_/NF pieces were separately put in two porcelain boats. The porcelain boat of sodium hypophosphite was presented at its upside in a tube furnace. The thermal decomposition process was conducted in the tube furnace at 400 °C for 2 h with a heating rate of 3 °C/min. Pure N_2_ atmosphere acted as the protecting atmosphere. PH_3_ can be produced during the thermal decomposition process of sodium hypophosphite. And Co(OH)_2_/NF was transformed into cobalt phosphide with the assistance of PH_3_. After being cooled to room temperature, the as-obtained sample was marked as Co-P-2.

### 3.4. Material Characterization

The phase composition of these electrocatalysts was characterized via X-ray Diffraction (XRD, a Bruker D8AA25 diffraction, Bruker AXS GMBH, Karlsruhe, Germany). The morphologies of as-prepared electrodes were examined using scanning electron microscopy (SEM, Carl Zeiss Management Co., Ltd., Jena, Germany). The surface elemental compositions and states were analyzed by an X-ray photoelectron spectrometer (XPS, Thermo Scientific K-Alpha, Waltham, MA, USA), and FTIR spectrum via a Shimadsu 4600 spectrometer (JASCO, Tokyo, Japan). The structure change in obtained catalysts before and after HER and OER is confirmed with Raman spectra (inVia confocal Micro Raman Spectroscopy (RTS2)).

### 3.5. OER Measurements

The electrochemical properties of as-prepared samples were measured with linear sweep voltammetry (LSV), cyclic voltammetry (CV), electrochemical impedance spectroscopy (EIS) and chronopotentiometry (CP) measurements using an electrochemical work station (CHI 760E, Chenhua Instruments, Shanghai, China). A three-electrode system was employed in 1 M KOH electrolyte with as-prepared samples as the working electrode, Pt plate as the counter electrode, and Hg/HgO electrode as the reference electrode, respectively. Owing to the alkaline electrolyte and long-term stability test, the Hg/HgO electrode was employed as a reference electrode. LSV was conducted at a scan rate of 5 mV/s to evaluate HER (80% *iR* correction) and OER (100% *iR* correction) activity. The CV curves tested with various scan rates from 50 to 200 mV/s in the non-faradaic region (0.7–0.8 V vs. RHE) were selected to calculate the double-layer capacitance (*C_dl_*) and the electrochemical active surface area (ECSA). The long-term stability of the electrocatalyst was measured by a CP test at 10 mA/cm^2^ for HER (24 h), 50 mA/cm^2^ for OER (24 h), and 10 mA/cm^2^ for overall water splitting (32 h), respectively. Electrochemical impedance spectroscopy (EIS) was applied at the potential of 1.5 V vs. RHE in the frequency range of 0.1–100,000 Hz. All potentials were converted into potential versus RHE. And the overpotential (*η*) at certain current density can be calculated as *η* = *E*_RHE_ − 1.23 V.

## 4. Conclusions

In summary, we successfully electrodeposited a needle-like Co-P-1 nanoarray on NF as an efficient bifunctional electrocatalyst for HER and OER. For HER performance, Co-P-1 expresses a low overpotential of 85 mV to deliver 10 mA/cm^2^. For OER performance, Co-P-1 possesses a low overpotential of 294 mV and a Tafel slope of 60 mV/dec to obtain 50 mA/cm^2^ with a good long-term stability (24 h). In addition, Co-P-1 also transforms into CoOOH after the OER process. The potential of 1.60 V is found to achieve a current density of 10 mA/cm^2^ for overall water splitting, comparable to the reported Pt/C/NF||RuO_2_/NF. The excellent electrocatalytic performance can be attributed to the needle-like structure with plentiful active sites, accelerated charge transfer and evolved bubbles’ release. The facile electrodeposition for bifunctional electrocatalysts supplies the potential for large-scale water-splitting devices.

## Data Availability

The data presented in this study are available on request from the corresponding author.

## Supplementary Materials

The following supporting information can be downloaded at https://www.mdpi.com/article/10.3390/molecules28166101/s1. Figure S1: XRD pattern of electrodeposited Co-P-1; Table S1: HER activity comparison with other cobalt-based electrocatalysts; Table S2: OER activity comparison with other cobalt-based electrocatalysts; Figure S2: (a) HER and (b) OER polarization curves without *iR*-correction of these catalysts; Figure S3: (a) CV curves in the potential range of −0.5–0.5 V vs. RHE, and (b–d) CV curves of NF, Co(OH)_2_/NF, and Co-P-2/NF at various scan rates; Figure S4: Nyquist plots of these catalysts fitted with inserted equivalent circuit; Table S3: Parameters fitted with equivalent circuit model; Figure S5: (a) Initial SEM image and (b) SEM image after 24 h OER test of Co-P-1. References [16,19,41,42,43,44,45,46] are cited in the supplementary materials.

## Sample Availability

Samples of Co(OH)_2_, Co-P-1, and Co-P-2 are available from the authors.
