# Supplementary material for: Facile Electrochemical Synthesis of Bifunctional Needle-like Co-P Nanoarray for Efficient Overall Water Splitting
Source: Molecules. 2023 Aug 17;28(16):6101. doi: 10.3390/molecules28166101 (PMC10459374; doi:10.3390/molecules28166101)
Supplement: Supplementary file 1 [file molecules-28-06101-s001.zip › molecules-2551421-supplementary.pdf]

Supporting Information

Facile Electrochemical Synthesis of Bifunctional Needle-like Co-P Nanoarray for Efficient Overall Water Splitting

Xiong He [1], Jiayang Cai [2], Jie Zhou [1], Qiyi Chen [1], Qijun Zhong [1], Jinghua Liu [1,*], Zijun Sun [1], Dezhi Qu [2] and Yudong Li [3,*]

[1] School of Electronic Engineering, Liuzhou Key Laboratory of New Energy Vehicle Power Lithium Battery, Guangxi Engineering Research Center for Characteristic Metallic Powder Materials, Guangxi University of Science and Technology, Liuzhou 545000, China; hexiong@gxust.edu.cn (X.H.); 17586600924@163.com (J.Z.); 15296323062@163.com (Q.C.); zqj17878906682@163.com (Q.Z.); sunzijun@gxust.edu.cn (Z.S.)

[2] Guangxi Key Laboratory of Green Processing of Sugar Resources, College of Biological and Chemical Engineering, Guangxi University of Science and Technology, Liuzhou 545006, China; cjy193677464@163.com (J.C.); qudezhi199166@gxust.edu.cn (D.Q.)

[3] Key Laboratory of Bio-Based Material Science & Technology, Northeast Forestry University, Harbin 150090, China

* Correspondence: liujinghua@gxust.edu.cn (J.L.); lydlnn0000@163.com (Y.L.)

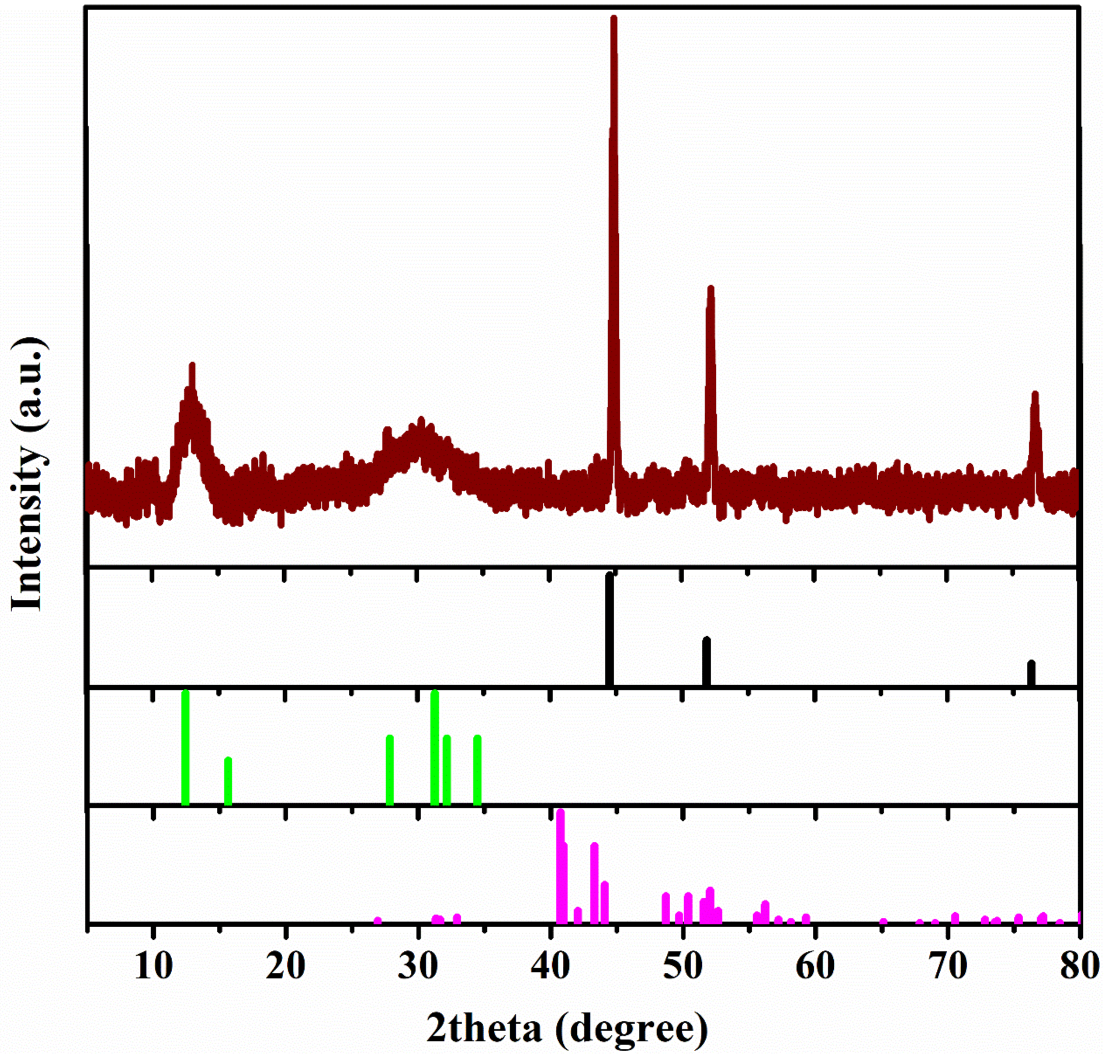

Figure S1 XRD pattern of electrodeposited Co-P-1.

Table S1 HER activity comparison with other cobalt-based electrocatalysts

Electrocatalysts	Overpotential	Tafel slope	Reference
**Co-P-1**	**86 mV at 10 mA/cm^2**	**60 mV/dec**	**This work**
NiCoP	120 mV at 10 mA/cm^2	51 mV/dec	[1]
Co-P	176.5 mV at 10 mA/cm^2 (0.5 M H_2SO_4)	80 mV/dec	[2]
C-CoP	173 mV at 10 mA/cm^2	63 mV/dec	[3]
CeO_x/CoP	117 mV at 10 mA/cm^2	97 mV/dec	[4]
CoP/C	140 mV at 10 mA/cm^2	109 mV/dec	[5]
CoP	86.6 mV at 10 mA/cm^2	71 mV/dec	[6]

Table S2 OER activity comparison with other cobalt-based electrocatalysts

Electrocatalysts	Overpotential	Tafel slope	Reference
**Co-P-1**	**294 mV at 50 mA/cm^2**	**60 mV/dec**	**This work**
NiCoP	276 mV at 10 mA/cm^2	111 mV/dec	[1]
C-CoP	333 mV at 10 mA/cm^2	71 mV/dec	[3]
CeO_x/CoP	264 mV at 10 mA/cm^2	82 mV/dec	[4]
CoP/C	250 mV at 10 mA/cm^2	138 mV/dec	[5]
$Co(OH)_2$	322 mV at 10 mA/cm^2	96 mV/dec	[7]
MnOOH/CoOOH	313 mV at 10 mA/cm^2	87 mV/dec	[8]

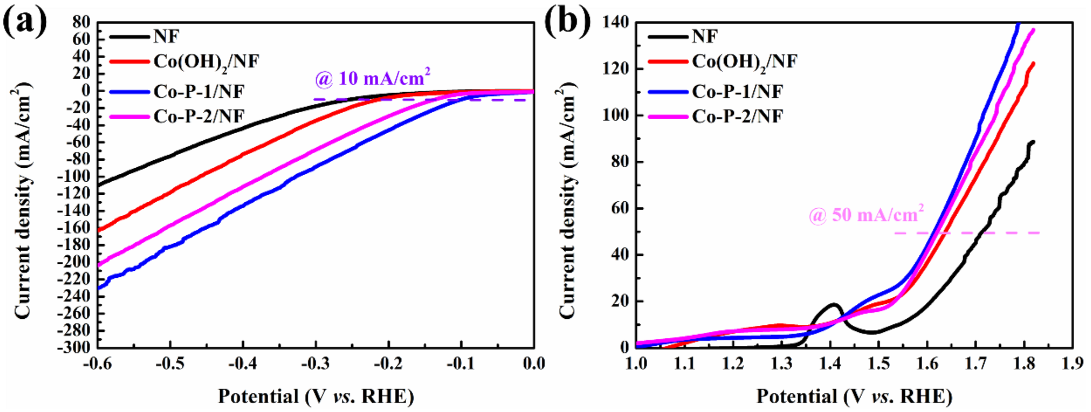

Figure S2 (a) HER and (b) OER polarization curves without *iR*-correction of these catalysts.

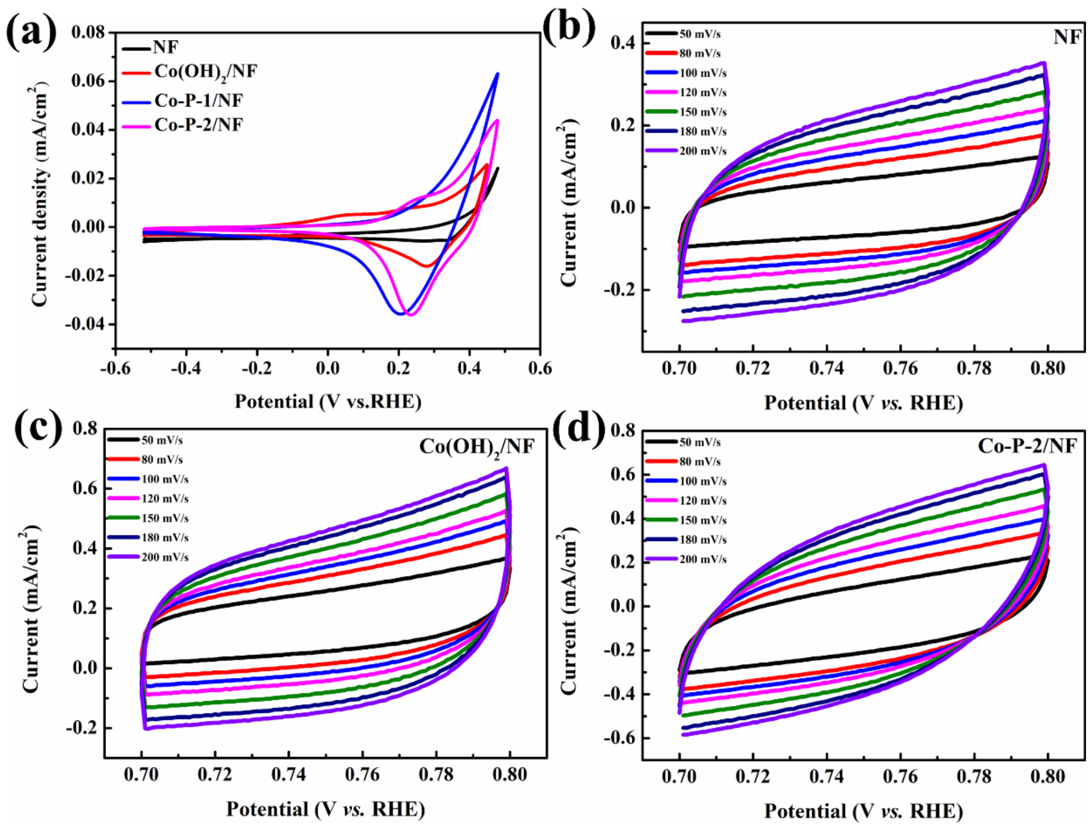

Figure S3 (a) CV curves in the potential range of -0.5-0.5 V vs. RHE, and (b-d) CV curves of NF, $Co(OH)_2$ /NF, and Co-P-2/NF at various scan rates.

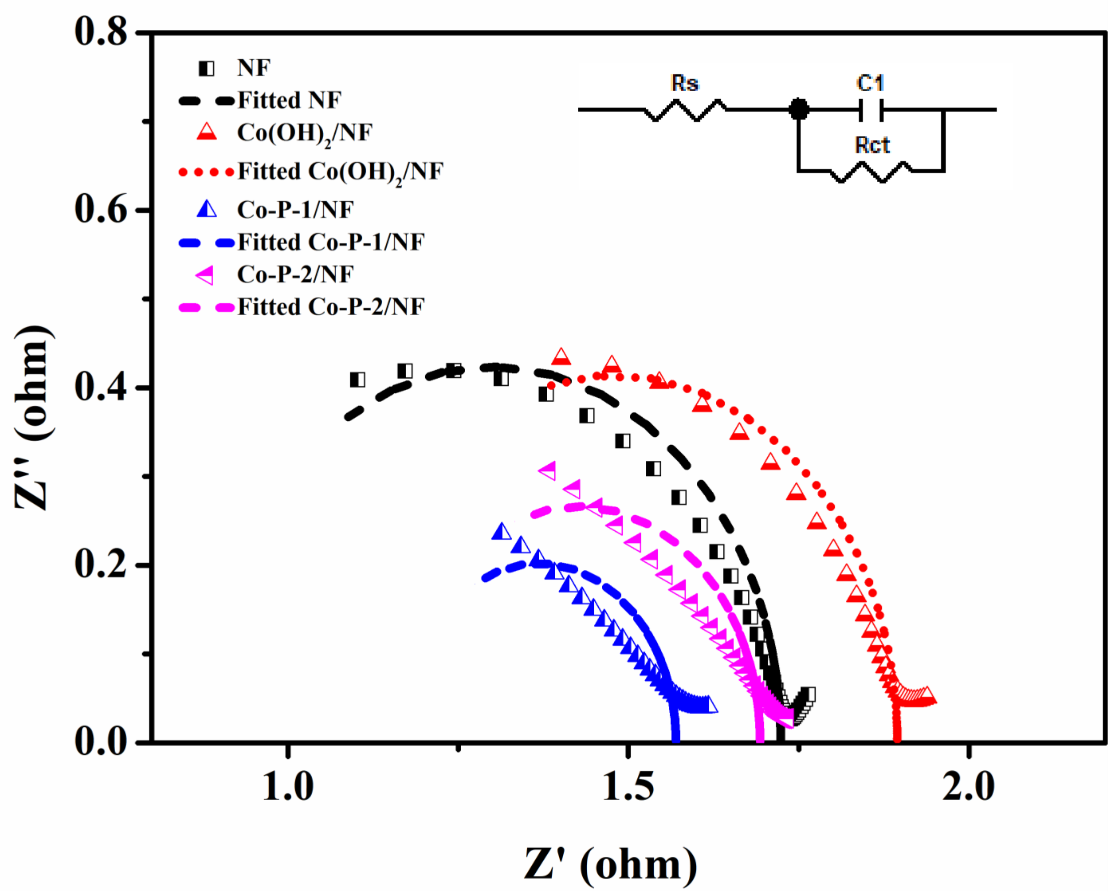

Figure S4 Nyquist plots of these catalysts fitted with inserted equivalent circuit model

Table S3 Parameters fitted with equivalent circuit model

Electrocatalysts	*Rs* (Ω)	R_{ct} (Ω)
NF	0.87	0.85
$Co(OH)_2$/NF	1.07	0.83
Co-P-1/NF	1.16	0.41
Co-P-2/NF	1.15	0.54

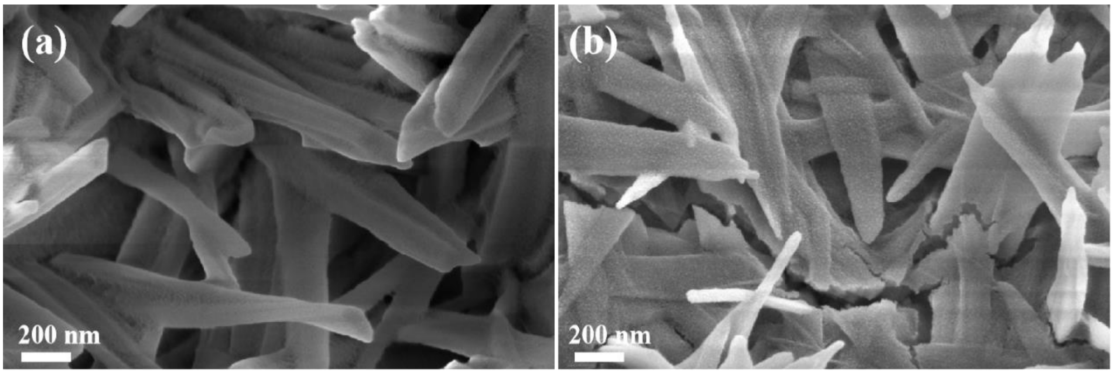

Figure S5 (a) Initial SEM image and (b) SEM image after 24 h OER test of Co-P-1.

References

1. Sheng, M.; Yang, Y.; Bin, X.; Que, W., One-Step Electrochemical Synthesis and Surface Reconstruction of NiCoP as an Electrocatalyst for Bifunctional Water Splitting. *Materials* **2023,** 16, (4), 1529.
2. Kim, J.; Jang, Y. J.; Jang, Y. H., Electrodeposition of Stable Noble-Metal-Free Co-P Electrocatalysts for Hydrogen Evolution Reaction. *Materials* **2023,** 16, (2), 593.
3. Li, W.; Cheng, G.; Sun, M.; Wu, Z.; Liu, G.; Su, D.; Lan, B.; Mai, S.; Chen, L.; Yu, L., C-CoP hollow microporous nanocages based on phosphating regulation: a high-performance bifunctional electrocatalyst for overall water splitting. *Nanoscale* **2019,** 11, (36), 17084-17092.
4. Zhang, T.; Wu, X.; Fan, Y.; Shan, C.; Wang, B.; Xu, H.; Tang, Y., Hollow CeOx/CoP Heterostructures Using Two-dimensional Co−MOF as Template for Efficient and Stable Electrocatalytic Water Splitting. *ChemNanoMat* **2020,** 6, (7), 1119-1126.
5. Li, X.; Qian, X.; Xu, Y.; Duan, F.; Yu, Q.; Wang, J.; Chen, L.; Dan, Y.; Cheng, X., Electrodeposited cobalt phosphides with hierarchical nanostructure on biomass carbon for bifunctional water splitting in alkaline solution. *Journal of Alloys and Compounds* **2020,** 829, 154535.
6. Guo, P.; Wu, Y.-X.; Lau, W.-M.; Liu, H.; Liu, L.-M., Porous CoP nanosheet arrays grown on nickel foam as an excellent and stable catalyst for hydrogen evolution reaction. *International Journal of Hydrogen Energy* **2017,** 42, (44), 26995-27003.
7. De Silva, O.; Singh, M.; Mahasivam, S.; Mahmood, N.; Murdoch, B. J.; Ramanathan, R.; Bansal, V., Importance of Phase Purity in Two-Dimensional β-Co(OH)2 for Driving Oxygen Evolution. *ACS Applied Nano Materials* **2022,** 5, (9), 12209-12216.
8. Cui, M.; Zhao, H.; Dai, X.; Yang, Y.; Zhang, X.; Luan, X.; Nie, F.; Ren, Z.; Dong, Y.; Wang, Y.; Yang, J.; Huang, X., Promotion of the Electrocatalytic Oxygen Evolution Reaction by Chemical Coupling of CoOOH Particles to 3D Branched γ-MnOOH Rods. *ACS Sustainable Chemistry & Engineering* **2019,** 7, (15), 13015-13022.
